# Survival-time dependent increase in neuronal IL-6 and astroglial GFAP expression in fatally injured human brain tissue

**DOI:** 10.1038/s41598-019-48145-w

**Published:** 2019-08-15

**Authors:** Florian Trautz, Heike Franke, Simone Bohnert, Niels Hammer, Wolf Müller, Ruth Stassart, Rexson Tse, Johann Zwirner, Jan Dreßler, Benjamin Ondruschka

**Affiliations:** 1 Institute of Legal Medicine, Medical Faculty University of Leipzig, Leipzig, Germany; 2 Rudolf Boehm Institute of Pharmacology and Toxicology, Medical Faculty University of Leipzig, Leipzig, Germany; 3 Institute of Forensic Medicine, University of Würzburg, Würzburg, Germany; 4 Department of Anatomy, University of Otago, Dunedin, New Zealand; 5 Department of Orthopedic and Trauma Surgery, University Hospital of Leipzig, Leipzig, Germany; 6 Fraunhofer IWU, Dresden, Germany; 7 Department of Neuropathology, University Hospital of Leipzig, Leipzig, Germany; 8 Department of Forensic Pathology, LabPLUS, Auckland City Hospital, Auckland, New Zealand

**Keywords:** Diagnostic markers, Outcomes research, Cell death in the nervous system

## Abstract

Knowledge on trauma survival time prior to death following a lethal traumatic brain injury (TBI) may be essential for legal purposes. Immunohistochemistry studies might allow to narrow down this survival interval. The biomarkers interleukin-6 (IL-6) and glial fibrillary acidic protein (GFAP) are well known in the clinical setting for their usability in TBI prediction. Here, both proteins were chosen in forensics to determine whether neuronal or glial expression in various brain regions may be associated with the cause of death and the survival time prior to death following TBI. IL-6 positive neurons, glial cells and GFAP positive astrocytes all concordantly increase with longer trauma survival time, with statistically significant changes being evident from three days post-TBI (p < 0.05) in the pericontusional zone, irrespective of its definite cortical localization. IL-6 staining in neurons increases significantly in the cerebellum after trauma, whereas increasing GFAP positivity is also detected in the cortex contralateral to the focal lesion. These systematic chronological changes in biomarkers of pericontusional neurons and glial cells allow for an estimation of trauma survival time. Higher numbers of IL-6 and GFAP-stained cells above threshold values in the pericontusional zone substantiate the existence of fatal traumatic changes in the brain with reasonable certainty.

## Introduction

Traumatic brain injury (TBI) is still a major issue in public health^1^, and one of the most frequent causes of violent death^2–4^. Determining TBI as the cause of death at post-mortem examination (autopsy and/or post-mortem computed tomography) is relatively easy and commonly made in the forensic routine. Although it is challenging to estimate the survival time after TBI, i.e. the duration between the head injury and the time of death, it may be essential for legal purposes.

To date, the estimation of TBI survival time is investigated using histology, immunohistochemistry^5–8^, immunocytochemistry^9^, immunoblotting^10,11^, biochemistry^12–14^ and gene expression analyses^15,16^. However, the underlying pathophysiological cascade following TBI is not entirely understood^17^. TBI causes immediate local neuronal and glial cell death (necrosis), axonal rupture, and glial activation with subsequent neurotoxicity^18^. This initiates multiple complex pathways between glial cells and neurons forming a cascade of secondary brain injuries, such as brain edema, metabolic disturbance, oxidative stress^19,20^ and inflammatory changes as an acute phase response^17,21^. These reactions aim to remodel and restore brain plasticity^22,23^.

Forensic neurotraumatology utilizes biomarkers or proteins with specificity for structures of the central nervous system (CNS) to illustrate the changes in cellular mechanisms after brain tissue damage, either following an ischemic/hypoxic or a traumatic event^7,24–26^. In order to add to the published data for TBI wound age in cases where the survival time was unknown, we examined brain tissue samples from autopsy cases with lethal TBI and compared them to cardiovascular causes of death immunohistochemically and via immunofluorescence for interleukin-6 (IL-6) and glial fibrillary acidic protein (GFAP).

IL-6 as a well-known cytokine is a small glycoprotein with a molecular weight less than 30 kDa^27^. It is predominantly expressed by neurons and glial cells in the CNS and mediates complex reactions such as the pro-inflammatory response^28^, but also has neuroprotective potential with trophic, anti-apoptotic and anti-inflammatory characteristics^29,30^. These contrary effects seem to depend on the local IL-6 concentration^31–33^, which increases under certain circumstances such as TBI, subarachnoid hemorrhage or CNS infection^27^. Given its initiator function of acute phase response, it is considered as an early TBI marker^34^. GFAP is one of the most-widely studied proteins in neuropathology. This protein is a type III intermediate filament involved in maintaining the blood brain barrier. GFAP also provides stability in the astrocytes throughout the CNS and is essential for reactive processes such as astrogliosis and glial scar formation^35^.

Both IL-6 and GFAP are well-established biomarkers used in living patients with TBI to confirm and predict the existence and severity of the brain injuries and to determine the potential neurological outcome in trauma patients^36–40^. Furthermore, both markers are established for post-mortem biochemistry, with promising results for cerebrospinal fluid investigations in assessing the severity of brain damage due to a lethal TBI^13,14^. To our best knowledge on human TBI research, intracellular IL-6 has to date not been investigated immunohistochemically, and GFAP has mostly only been investigated in cortical samples; both proteins were therefore chosen for the presented forensic study, in particular, to estimate the survival time in a lethal TBI. This study compared the immunohistochemical and immunofluorescence profile for intracellular IL-6 and GFAP in brain tissue between lethal TBI (with well documented survival time) and cardiovascular causes of death, to determine whether the level of positive reactivity of both proteins in neurons or glial cells of various brain regions is associated with the cause of death and the survival time after fatal TBI.

## Results

### Demographic data

54 TBI cases were identified in the study (for selection process see Supplemental Fig. 1), and the pericontusional zone (PCZ, n = 54), the contralateral cortex (n = 53), the CA4 region of the hippocampus (n = 41) and the cerebellum (n = 47) were examined. These results from TBI were compared to 21 controls with cardiovascular causes of death (sampled for frontal cortex, hippocampus and cerebellum). All deceased were of Caucasian descent. The basic comparison between the TBI and control cases resulted in sex- and post-mortem interval (PMI)-matching (p = 0.61 and p = 0.14, respectively). The age for the deceased in trauma cases of the acute death group was significantly younger compared to the subacute phase and the control cases (p = 0.03, for details see Supplemental Fig. 2). Table 1 shows the case characteristics according to their survival time category and causes of death. Isolated TBI or severe TBI accompanied by multiple other injuries to the body were the main causes of death in both acute and subacute traumas. In those cases with a longer survival time, the number of fatal traumatic brain edema increased over time and secondary lethal complications such as thromboembolisms and inflammatory changes dominated.

**Table 1:** Overview of the case characteristics according to their survival time category and cause of death.

Group	Characteristics								
	Sex		Age		Survival time		Post-mortem interval		Cause of death
	*Male*	*Female*	*Range*	*Median*	*Range*	*Median*	*Range*	*Median*	
**TBI group**									
Acute Death (n = 26)	19	7	18–75	44	0 h–2 h	0.25 h	5 h–117 h	44 h	Isolated TBI (n = 8), polytrauma (n = 12), traumatic brain edema (n = 3), aspiration (n = 2), hemorrhage shock (n = 1)
Subacute Death (n = 16)	11	5	23–85	66	2 h–60 h	5.5 h	31 h–144 h	70 h	Isolated TBI (n = 8), polytrauma (n = 1), traumatic brain edema (n = 4), aspiration (n = 2), hemorrhage shock (n = 1)
Delayed Death (n = 12)	10	2	26–81	57	74 h–3477 h	277 h	22h–132 h	78 h	Traumatic brain edema (n = 5), pulmonary thromboembolism (n = 2), pneumonia (n = 3), septic multi-organ failure (n = 2)
**Control group**									
Control (n = 21)	13	8	27–91	64	none	—	26 h–139 h	70 h	Acute myocardial infarction (n = 12), cardiac insufficiency (n = 4), ruptured aortic aneurysm (n = 4), pulmonary thromboembolism (n = 1)
P value	0.61		0.03		no calculation		0.14		

Please note, that survival time ‘0 h’ was quoted for acute deaths after TBI for cases with only minutely survival. TBI, traumatic brain injury.

No illicit drugs were detected when analyzed (toxicological results were available in 2/3 of the cases, n = 50 of 75 in total). Blood alcohol levels above 0 were observed in four TBI cases with acute and subacute death (range 0.73 to 3.29 mg/g) and two controls (0.21 and 1.08 mg/g).

**Figure 1:**
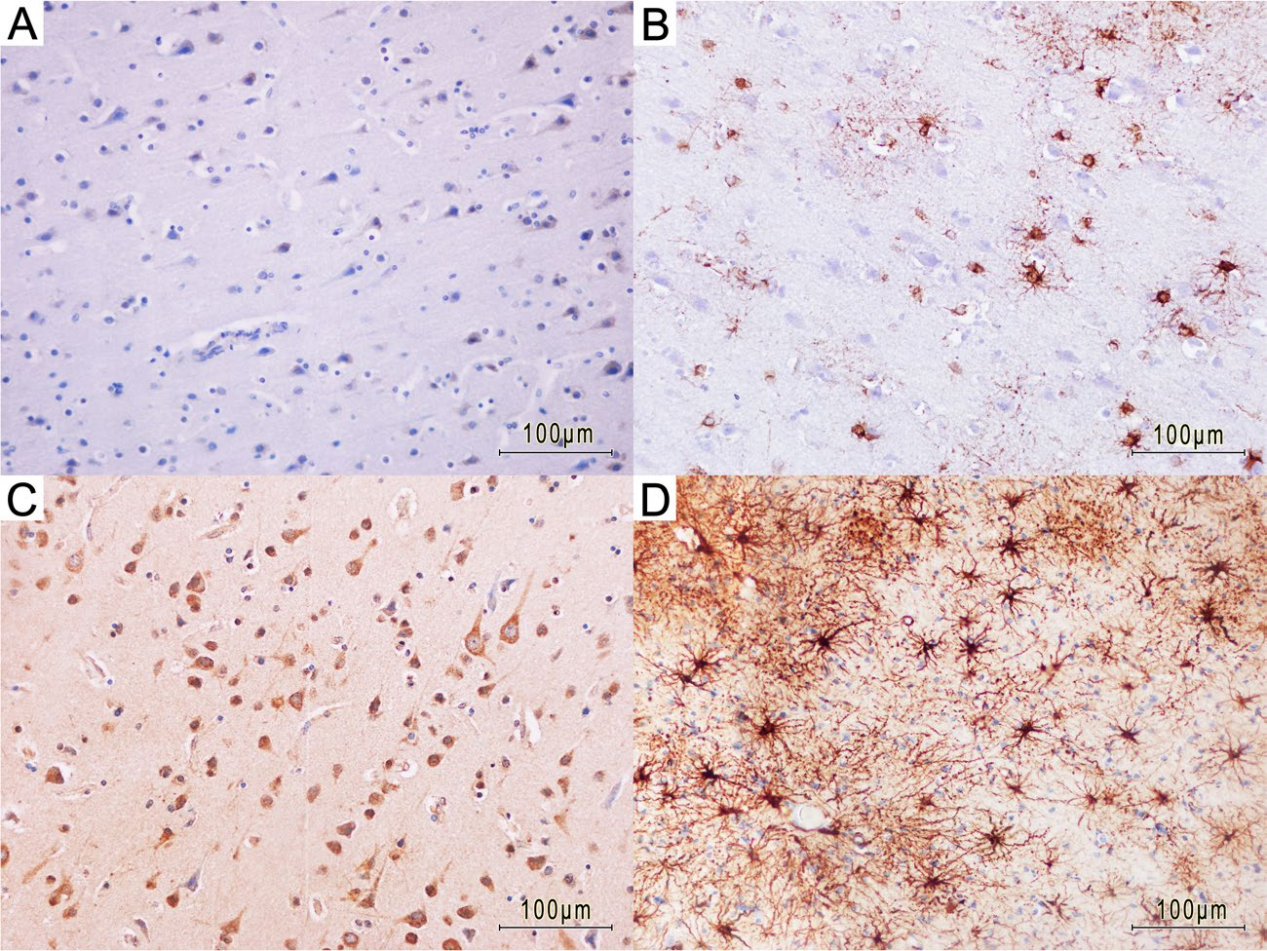
Examples of the staining evaluation for interleukin-6 (IL-6; A + C) and glial fibrillary acidic protein (GFAP; B + D) for different survival times. A traumatic brain injury case with a survival time of 2.5 hours with minutely IL-6 immunopositivity in neurons (**A**) and some more GFAP-positive astrocytes (**B**) in the pericontusional zone. IL-6-positive staining in neurons and glial cells (**C**) and an astrogliosis containing numerous GFAP-positive astrocytes (**D**) surrounding a cortical contusion (not shown in the exemplified pictures) in a traumatic brain injury case with a survival time of 5 days.

Figure 1 displays typical staining results for intracellular IL-6 and GFAP and their cellular appearance for two different trauma survival times (2.5 hours vs. 5 days).

### Post-traumatic IL-6 positive neuron ratio depends on age but total glial cell count is unaffected by age, sex and PMI

IL-6 positive neuron percentage had a moderate positive correlation with age at death in the TBI samples in PCZ (r = 0.34) and the contralateral cortex (r = 0.35). This circumstance was not observed for the hippocampus and the cerebellum samples. The IL-6 positive count of glial cells remained unaffected by age, sex and PMI in all cases (Table 2).

**Table 2:** Correlative comparisons between positive cellular expressions on neurons and glial cells for both antibodies used interleukin-6 (IL-6) and glial fibrillary acidic protein (GFAP) compared to the area of investigation, age, sex, post-mortem interval (PMI) and grading of GFAP staining intensity of the cases included.

Group	Marker	Cell population	Localization	Age (in y)	Sex	PMI (in h)	Grading
Traumatic brain injury	IL-6	Neuronal positive percentage	PCZ	0.34 (*)	−0.13	0.00	
			CLC	0.35 (*)	−0.06	−0.04	
			HC	0.27	−0.03	−0.22	
			CB	0.07	−0.01	0.06	
		Glial positive numbers	PCZ	0.08	0.00	−0.06	
			CLC	0.17	−0.09	−0.08	
			HC	0.08	−0.01	0.03	
			CB	0.12	−0.18	0.00	
	GFAP	Astrocytes positive numbers	PCZ	0.03	0.01	−0.13	0.38 (*)
			CLC	0.28	0.01	−0.02	0.35 (*)
			HC	0.07	−0.14	0.22	0.45 (*)
			CB	−0.12	−0.02	−0.01	0.45 (*)
Control	IL-6	Neuronal positive percentage	PFC	−0.28	0.18	0.06	
			HC	−0.41	0.19	0.15	
			CB	−0.27	0.05	−0.21	
		Glial positive numbers	PFC	−0.05	−0.08	0.26	
			HC	−0.10	−0.41	−0.03	
			CB	−0.26	0.18	−0.15	
	GFAP	Astrocytes positive numbers	PFC	−0.02	0.59 (*)	0.17	0.56 (*)
			HC	0.26	0.51	0.32	0.61 (*)
			CB	−0.19	0.64 (*)	0.11	0.61 (*)

PCZ, pericontusional zone; CLC, contralateral cortex; HC, hippocampus formation; CB, cerebellum; y, years; h, hours; *p < 0.05.

The number of GFAP positive astrocytes did not correlate with age, sex and PMI in TBI cases, whereas statistically significant positive correlations were found for GFAP positive astrocytes in the uninjured prefrontal cortex (r = 0.59) and cerebellum (r = 0.64) in control cases with the sex of the deceased (Table 2).

### Positive staining of IL-6 neurons and glial cells as well as GFAP positive astrocytes increase with longer TBI survival time

**Figure 2:**
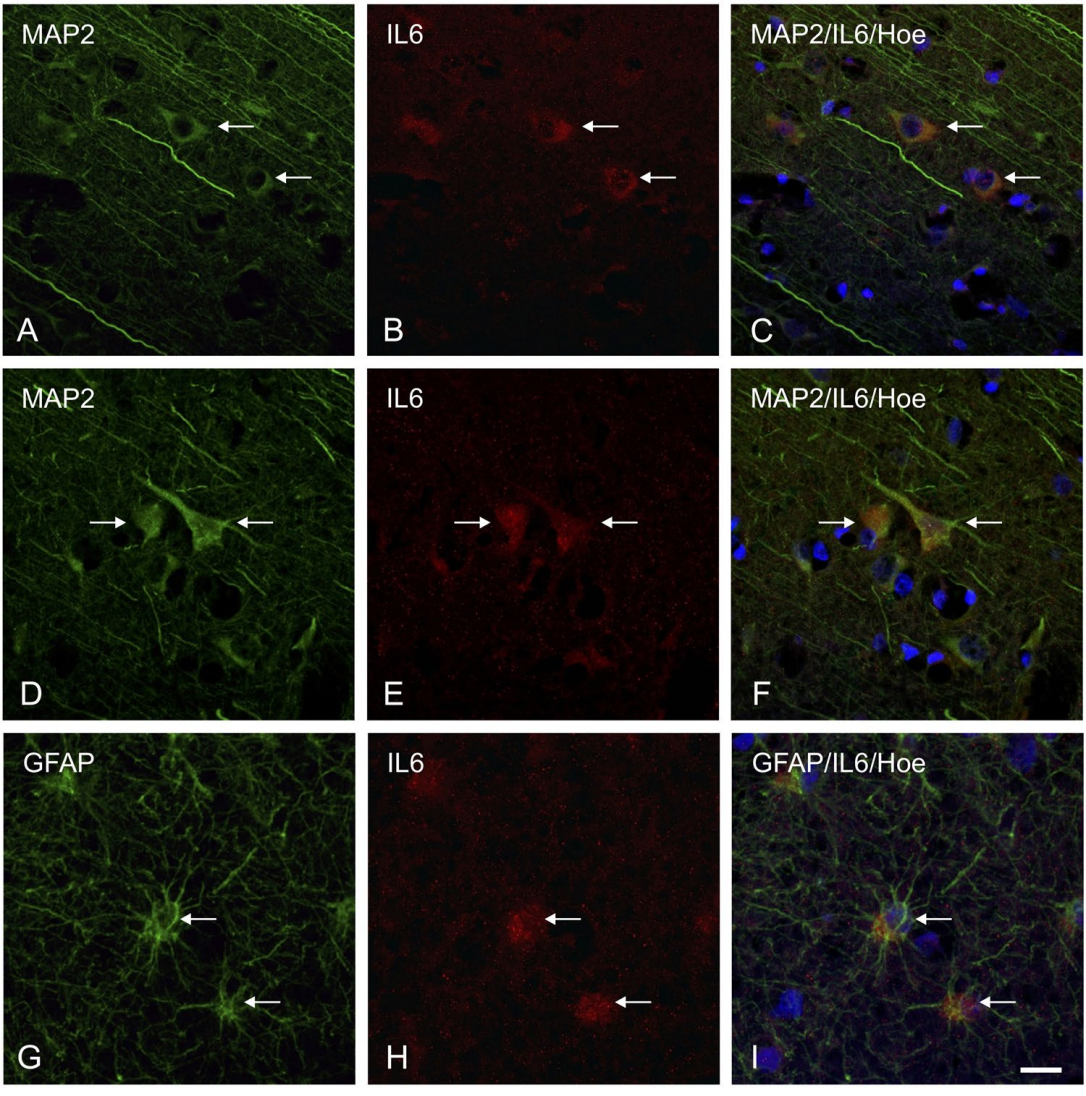
Double immunofluorescence in brain cortex samples of traumatic brain injury cases. Representative IL-6-positive stained neurons and astrocytes counterstained with MAP-2 (as a marker for neurons) and GFAP (as a marker for astrocytes) and Hoechst (Hoe)-positive cell nuclei using confocal microscopy. IL-6 and MAP-2 positive multipolar neurons (**A**–**C)** as well as in triangular neuronal cells **(D**–**F)** with double labeling (arrows). Astrocytic double labeling of IL-6 and GFAP (arrows) in vital glial cells (Hoe stained nuclei), **(G**–**I)**. Scale bars: 50 μm.

IL-6 positive cells were defined as staining positively for both neurons and glial cells. The existence of neuronal and glial IL-6 expression was proved positively using double immunofluorescence evaluations with typical marker staining for both neurons (by MAP-2) and astrocytes (by GFAP) (Fig. 2). Intracellular IL-6 showed more intense expression patterns in neurons than in glial cells. For both neurons and astrocytes, vital cells with intact nuclei (Hoechst counterstaining positive) has an IL-6 positive staining.

Positively stained neurons were counted in all brain regions in both TBI and controls, mostly in high numbers (highest count median in cortical PCZ = 87, interquartile range [IQR] 136), and also counted in hippocampal samples (median 81, IQR 172), with the lowest count median being in the cerebellar slides with 11, IQR 16); glial cells, however, were stained sparsely and mostly single cells only. GFAP was almost exclusively stained positively in astrocytes.

**Figure 3:**
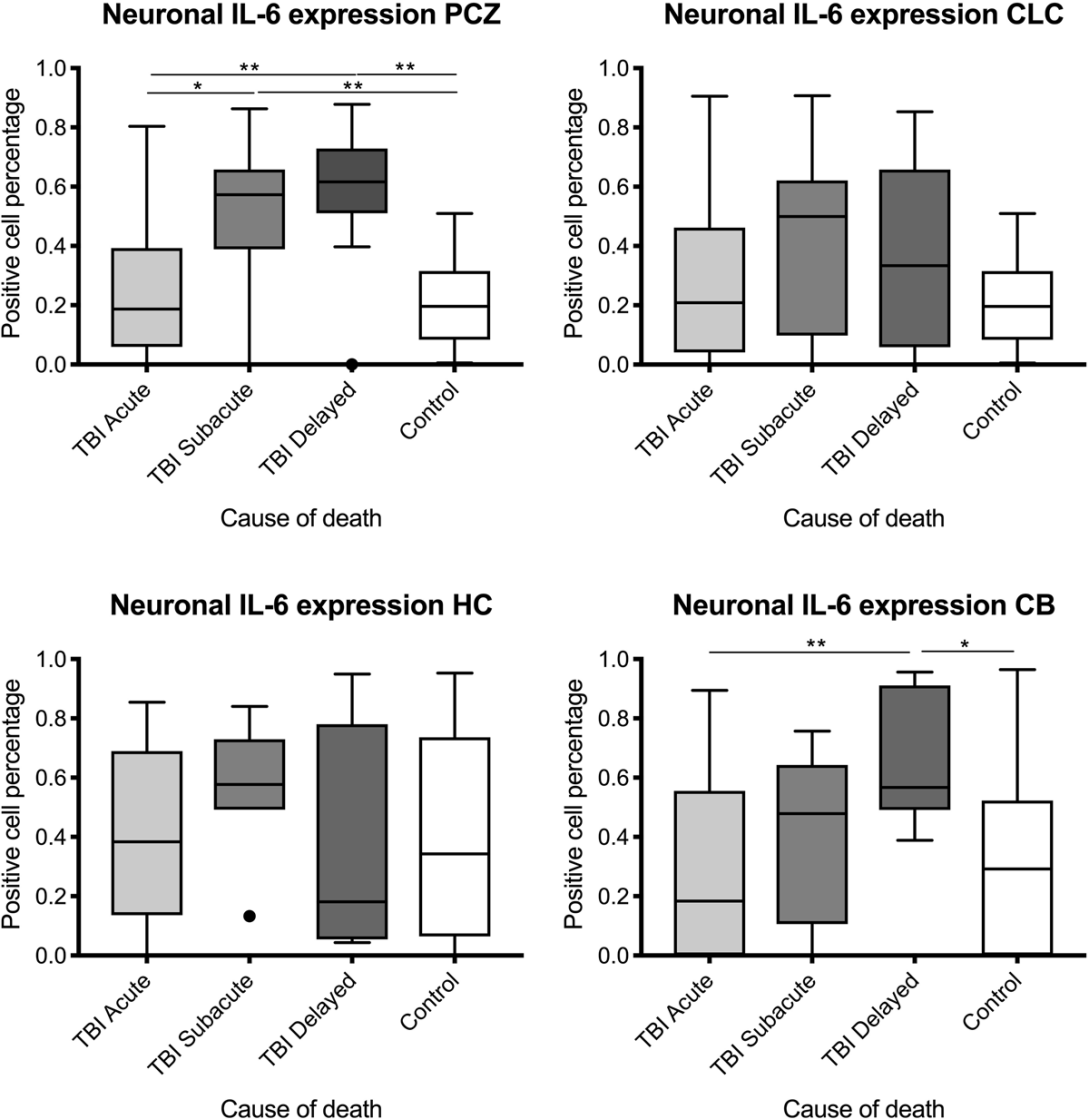
Box plot diagrams displaying the positive ratio of interleukin (IL-6)-positive neurons (counted in ten digital images at a 200x magnification) depending on the survival time of traumatic brain injury (TBI) fatalities compared to the controls. The outlines of the boxes indicate the 25% and 75% percentile, the solid black line the median. End of lines show the minima and maxima. Outliers (>1.5 interquartile range) are depicted as a bold point. PCZ, pericontusional zone; CLC, contralateral cortex; HC, hippocampus; CB, cerebellum. *p < 0.05; **p < 0.001 using Kruskal-Wallis test followed by *post hoc* Dunn’s test.

There were statistically significant differences between the IL-6 positive neuron ratio when comparing the survival time categories of TBI and controls in the PCZ (Kruskal-Wallis test: p < 0.0001) and the cerebellum (p = 0.0097), but not in the contralateral cortex (p = 0.1463) and the hippocampus (p = 0.5421, see Fig. 3).

For all four brain regions, the ratio of positive neuronal IL-6 expression did not reach statistical significance between acute TBI cases and controls. There was a non-significant increase in IL-6 neuron cell percentage in subacute death cases compared to cases with survival times of less than two hours, with a significant difference for the PCZ (p = 0.0111). The ratio increased in the PCZ and the cerebellum to reach a statistically significant level between delayed death cases after TBI and controls (p = 0.0018 for the PCZ, p = 0.0250 for the cerebellum).

**Figure 4:**
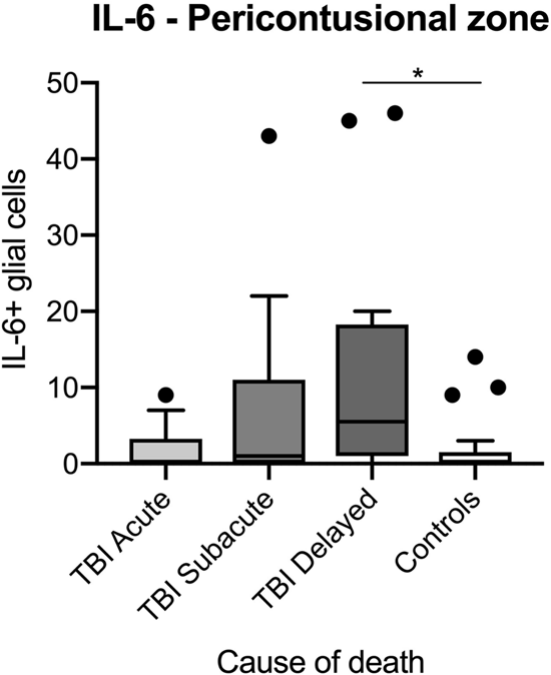
Box plot diagrams displaying the different total numbers of IL-6-positive glial cells (counted in ten digital images at a 200x magnification) depending on the survival time of traumatic brain injury (TBI) fatalities compared to the controls. The outlines of the boxes indicate the 25% and 75% percentile, the solid black line the median. End of lines show the minima and maxima. Outliers (>1.5 interquartile range) are depicted as a bold point. *p < 0.05 using Kruskal-Wallis test followed by *post hoc* Dunn’s test.

The number of IL-6 positive glial cells increased with longer survival times (p = 0.0182) with statistically significant differences between the count for delayed deaths after TBI and control cases (p = 0.025; see Fig. 4). Further comparison to other brain regions was not performed given the low number of IL-6 positive glial cells and their disseminated appearance.

The median value of GFAP positive astrocytes was higher in all brain regions in TBI compared to controls. The PCZ and contralateral cortex had overall lower GFAP positive astrocytes numbers compared to the hippocampus and the cerebellum.

**Figure 5:**
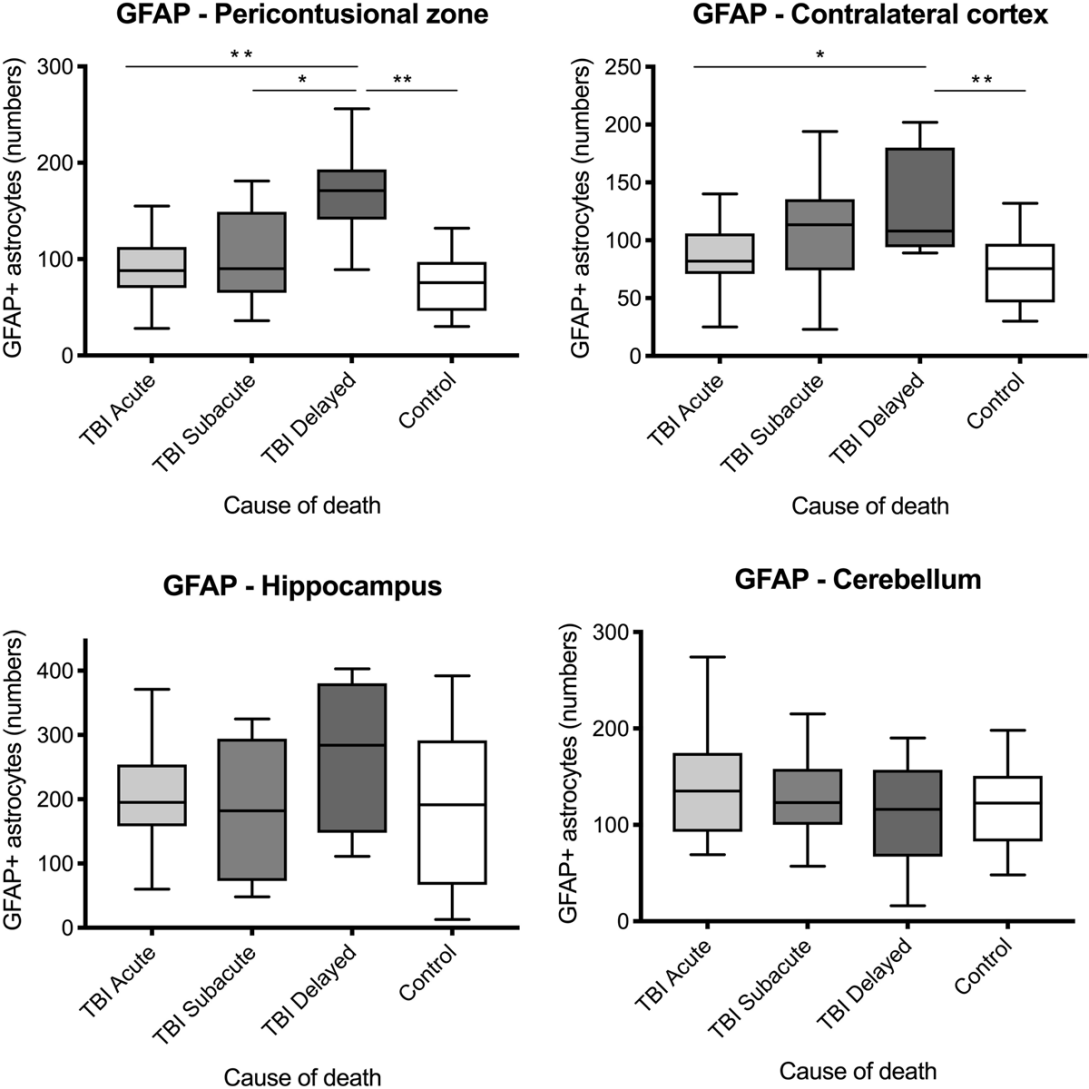
Box plot diagrams displaying the different total numbers of GFAP-positive astrocytes (counted in ten digital images at a 200x magnification) depending on the survival time of traumatic brain injury (TBI) fatalities compared to the controls. The outlines of the boxes indicate the 25% and 75% percentile, the solid black line the median. End of lines show the minima and maxima. *p < 0.05; **p < 0.001 using Kruskal-Wallis test followed by *post hoc* Dunn’s test.

For PCZ (p < 0.0001) and contralateral cortex samples (p = 0.0030) the difference of GFAP positive astrocytes between the survival time categories were statistically significant but did not reach statistical significance outside the cerebral cortex areas (p = 0.50 for hippocampus and p = 0.72 for cerebellum; see Fig. 5).

There were statistically elevated numbers of GFAP positive astrocytes in the PCZ and contralateral cortex after a post-TBI survival time longer than three days compared to the controls (p < 0.0001 for PCZ and p = 0.0046 for the contralateral cortex) and to acute deaths following TBI (p = 0.0009 for PCZ and p = 0.0424 for the contralateral cortex).

Comparing numbers of GFAP positive astrocytes and the staining intensities of the slides by a four-grade scoring system revealed statistically significant moderate to strong positive correlations in all brain regions investigated for both, TBI fatalities and controls (Table 2).

### Both IL-6 positive neurons and GFAP positive astrocytes correlate to the TBI survival time in the PCZ, but appear to be independent from the cortical localization of impact

**Figure 6:**
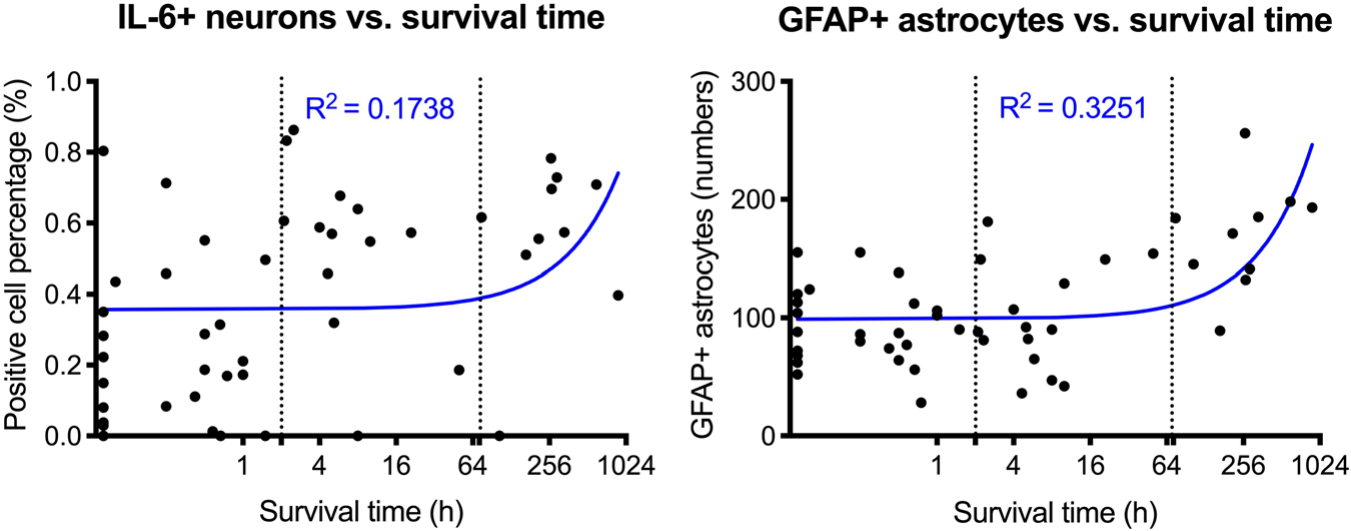
Scatter plots and corresponding blue regression lines depicting the association between IL-6 positive neurons (left) and GFAP positive astrocytes (right) in the pericontusional zone with the trauma survival time in hours (h), x axis presented as logarithm. For better visualization, the case with the longest survival time was not illustrated in the figure but was included in the statistical calculations. For IL-6: r = 0.27, p = 0.0309; for GFAP: r = 0.57, p < 0.001. The vertical dashed lines mark the time intervals between the survival time categories at 2 h and 72 h.

Scatter plots with regression lines demonstrate the moderate correlation of IL-6 positive neuronal percentage (r = 0.27, p = 0.0309) and the strong correlation of GFAP positive astrocytes (r = 0.57, p < 0.001) with the survival times of the single cases in the area surrounding the direct impact zone after trauma (Fig. 6).

The direct impact zones (coup injuries) were distributed randomly within the cerebral cortices but were located more frequently in the anterior compared to the posterior brain regions, irrespectively of the survival time. There were no statistically significant differences in the counting of IL-6 positive neurons and glial cells as well as GFAP positive astrocytes within the PCZ of different cortical regions (Table 3**)**.

**Table 3:** No significant influences between IL-6 and GFAP positive cell counts and the cortical area of a traumatic brain injury (TBI) impact within one survival time category were found.

TBI survival time	Contusion zone	Sample size	Test used	IL-6+ neurons	IL-6+ glial cells	GFAP+ astrocytes
Acute death	Frontal Tempoparietal Occipital	10 10 6	KW test	p = 0.61	p = 0.08	p = 0.53
Subacute death	Frontal Tempoparietal Occipital	8 8 0	MWU test	p = 0.52	p = 0.13	p = 0.51
Delayed death	Frontal Tempoparietal Occipital	4 6 2	KW test	p = 0.12	p = 0.38	p = 0.06

KW test, Kruskal-Wallis test; MWU test, Mann-Whitney U test.

Furthermore, a concordant and statistically significant increase was observed in both protein expression patterns in the PCZ of all trauma cases (Table 4).

**Table 4:** Correlative comparisons of IL-6 positive neuron ratio and GFAP positive astrocytes in the different brain regions investigated divided in traumatic brain injury (TBI) and control cases.

Case group	Localization	Spearmans 𝛠	P value
TBI	Pericontusional zone	0.41	0.0044
	Contralateral cortex	0.24	0.1071
	Hippocampus	−0.44	0.0377
	Cerebellum	0.04	0.7987
Control	Frontal cortex	0.13	0.6423
	Hippocampus	0.34	0.1360
	Cerebellum	0.09	0.7286

### Threshold values which may differentiate TBI from control cases

**Figure 7:**
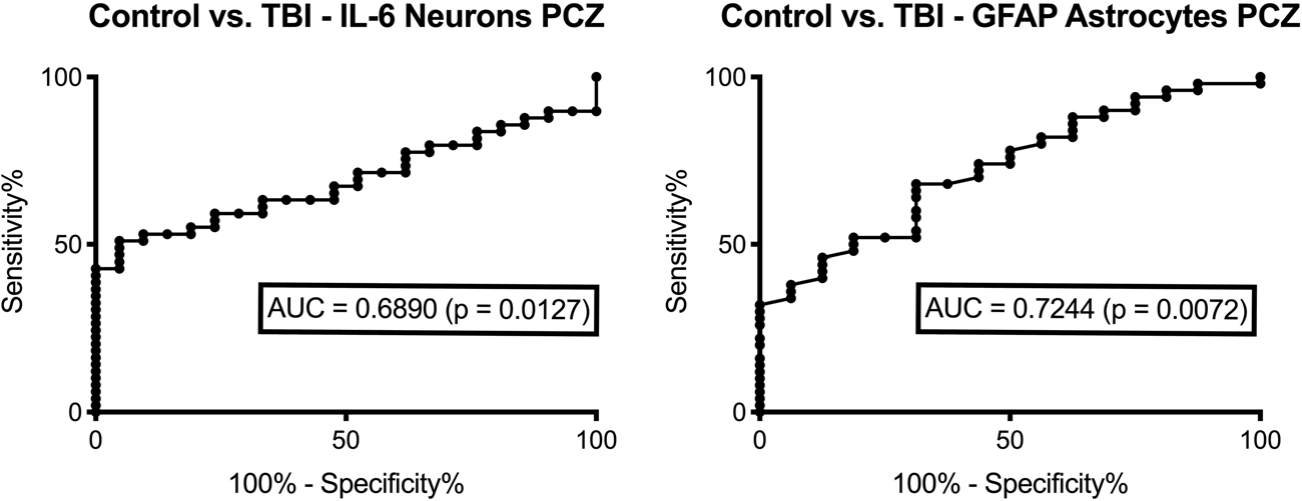
Receiver operating characteristic (ROC) curves for identification of the sensitivity, specificity and the area under the curve (AUC) of marker expressions in control vs. traumatic brain injury (TBI) cases presented for the analysis of the pericontusional zone (PCZ). Threshold values were calculated by conservative estimations for the differentiation between TBI and controls.

Based on receiver operating characteristic (ROC) curve analysis, TBI cases could be differentiated from cardiovascular control cases using immunohistochemical staining with intracellular IL-6 and GFAP of cortical samples (Fig. 7). Using the diagnostic accuracy of the applied method in its most conservative way, an IL-6 positive neuron ratio higher than 0.42 (sensitivity 51.0%, specificity 95.2%) substantiated the suspicion of an existing TBI in a positive likelihood ratio of 10.7. This positive threshold ratio was reached in the investigated TBI cases with survival times of at least 15 min, even in acute deaths after trauma. The threshold was more frequently reached in subacute TBI fatalities (10 of 16 cases) and delayed death periods (9 of 12 cases). There was only one single control case exceeding the threshold.

The ROC curve of GFAP astrocyte number in the PCZ demonstrated its discriminative character between TBI cases and controls. More than 106 GFAP positive astrocytes (sensitivity 46.0%, specificity 87.5%) were documented as a conservative threshold value with a positive likelihood ratio of 3.7.

This value was already reached in seven acute death cases after TBI (of 26 fatalities) but mostly in trauma survival times longer than three days (10 of 12 cases). Equal to intracellular IL-6, the threshold for GFAP was exceeded in only one case among the control group.

### The influence of ethanol consumption prior to TBI seems to be an irrelevant confounder

The few cases with positive blood alcohol content did not exceed both thresholds in the cortical samples (with the exception of one IL-6 positive neuronal ratio of 0.64 after 8 h TBI survival). The IL-6 positive neuronal percentage and GFAP positive astrocyte numbers were all distributed within the interquartile ranges of the results.

## Discussion

The object of the given study was to assess the usability of two CNS biomarkers for the forensic time since death estimation in TBI-related cases. Thus, the positivity of intracellular IL-6 and GFAP as neuronal and glial proteins was investigated in different brain regions related to the cause of death and survival time. This study showed that the IL-6 positive neuron ratio in the PCZ and the cerebellum, and the number of GFAP positive astrocytes in the PCZ and the contralateral cortex increased with longer trauma survival times with significantly higher values in cases with survival times of three days or more. We were able to establish conservative thresholds for immunohistochemical investigations of PCZ slides for the routine histological preparation for both proteins, allowing the differentiation between TBI and control cases of natural deaths. Furthermore, we found a positive correlation between positive IL-6 stained neurons and GFAP stained astrocytes in the PCZ. Conclusively, the main results indicate that both intracellular IL-6 and GFAP may serve as markers of a systemic CNS reaction during secondary events after TBI, with the most intensive changes in expression numbers observed in proximity to the injured area, the PCZ.

We decided to compare a secreted ‘functional’ acute phase protein, IL-6, together with a structure protein of astrocytes, GFAP, to improve the accuracy of wound age estimation after TBI, also by investigating cell changes distant from the PCZ to provide information about the generalized brain condition after TBI. Since IL-6 was shown to stimulate the differentiation of astrocytes^41,42^, there is at least an indirect link between these two proteins. Finally, both chosen markers are commercially available which more realistically enables usage in forensic daily routine then.

### Intracellular IL-6 is expressed in increasing numbers of neurons and glial cells in the PCZ depending on the survival time

Animal research demonstrated that IL-6 is expressed in neurons and glial cells with specific patterns depending on the investigated brain area^43–45^. Aniszewska *et al*.^45^ found up to 90% positive stained cells in the cortex, hippocampus and cerebellum as neurons, while positive microglia only made up around 8%. However, immunopositive astrocytes were mainly detected surrounding the ventricular system (95%), and also in the cerebellum in other studies^46^. This immunopositivity is also described in humans for neurons, glia and microglia^47–49^. Regarding the investigated brain regions in this given study, the determined distribution amongst positive cells seems to be comparable in human specimens. Although intracellular IL-6 staining in human is not well established yet, artifacts influencing the results are unlikely given the similar staining results in both the immunohistochemical and the immunofluorescent methods used in this study. IL-6 was clinically shown to be actively produced in the PCZ by an *in-situ* detection method of surgical samples in the delayed phase after TBI^50^, which is in good accordance with our post-mortem results. This allows for paracrine effects in the trauma surrounding area during brain repair.

IL-6 acts as a plurifunctional cytokine and pro-inflammatory agent but contains also anti-inflammatory and neuroprotective characteristics and thereby participates in neurogenesis, thus, influencing the function of neurons and glial cells^45,51^. IL-6 has been repeatedly shown to increase after TBI in living patients^27,34^ and post-mortem specimens^13,52^ within hours after TBI. Its presence in cerebrospinal fluid is associated with poor neurological outcomes^53^ and higher intracranial pressure after TBI^54^. IL-6 is the cytokine in highest concentrations in human cerebrospinal fluid after TBI^55^ and is described as a vital mediator of neuroinflammation^50^. Physiologically, IL-6 as a secreted protein is expressed at relatively low levels in CNS cells^56^ and its intracellular staining on tissue can be considered as a snapshot of the IL-6 protein production. Probably a larger portion can be found in extracellular brain regions, e.g. the cerebrospinal fluid. Detection and quantification therein were however not part of this study due to the methodological framework used but has been investigated biochemically by our research group already^13^.

The protein has been shown to promote neural and axonal growth, differentiation of glial and Schwann cells^41,42^, but can also cause neuronal death when increased in pathological conditions^31^. Previous research from the authors demonstrated a six-fold increase in IL-6 mRNA expression in cerebellar samples of frontal TBI compared to control cases with trauma survival times up to 18 h^16^, and increased IL-6 cerebrospinal fluid levels in fatal TBI cases^13^ forming the rationale for studying the immunohistochemical intracellular IL-6 characteristics. Increased IL-6 mRNA levels in human PCZ after TBI has been previously reported^57^. This study provided further evidence that, in the PCZ and the ipsilateral cerebellum, an increased neuronal IL-6 ratio at protein level correlates with increased trauma survival time.

To our knowledge, only one human study in literature has investigated IL-6 immunohistochemically in post-mortem brain slides. Wei *et al*.^58^ used cerebellar homogenates of twelve infants to investigate autism-related alterations of the CNS and found significantly increased IL-6 cell counts in the cerebellum in this cohort. Unfortunately, the authors did not present specific staining patterns making comparisons to our results impossible. However, IL-6 overexpression did not initiate apoptosis in the cerebellum^58^. Post-mortem immunohistochemistry research on intracellular IL-6 staining was generally not available for the cortical brain and hippocampal tissue and for TBI.

The temporal profile of intracerebral extracellular IL-6 concentrations sampled by microdialysis in neurosurgery were highest on the second day after TBI which subsequently decreased^27^, which matched our previous post-mortem cerebrospinal fluid results on IL-6^13^ and the reciprocal intracellular expression described here. Contrary to the clinical findings in cerebrospinal fluid, there was no association of sex with intracellular IL-6 expression in the brain tissue^27^. We demonstrated a correlation between the IL-6 neuron positive ratio and the age of the deceased. This is not surprising given that, firstly, the main IL-6 results showed increased neuron ratios in longer TBI survival times and these time spans were most often reached by older patients than the TBI deaths with only short survival. Secondly, derived from animal model, the cytokine expression in the brain regions investigated generally increased with age^59,60^.

Since no statistically significant change in IL-6 positive neurons was detected in the PCZ of acute death cases, it is suggested that immediate lethal brain dysfunction due to the primary impact is independent of early intracellular IL-6 related inflammatory changes for up to 15 minutes. The very low numbers of IL-6 stained glial cells in the cerebrum, hippocampus and cerebellum shown here were also described in mice^45^. Reasonably, each of the different IL-6 expressing cell phenotypes might react to other stimuli and transcript according to different (patho-)physiological processes.

An expansion of the cortical contusion may also occur several days after the initial trauma^61^ by a delayed intracerebral inflammatory response contributing to secondary brain damage^50^. The significant correlation between the IL-6 positivity in neurons and the GFAP positive astrocyte numbers in the context of trauma survival time indicates a synergistic pathway of post-TBI inflammation and cellular swelling with astrogliosis as part of the secondary changes. This knowledge about the chronological regulation of the neurotrophin-like IL-6^51^ might allow for pharmacological interventions in living patients to reduce secondary brain injury effects.

At present, one IL-6 antibody is commercially available (tocilizumab) for rheumatoid arthritis treatment only^62^. Experimentally, in a rodent TBI model, a depletion of the cytokine had exacerbated the outcome, whereas higher IL-6 rates were associated to a faster healing and recovery after TBI^51,63^.

Interestingly, clinical serum analyses have shown a potential immunosuppressive effect of positive blood alcohol concentrations in TBI patients resulting in reduced IL-6 levels in the blood^64^. However, the four investigated TBI cases in the given cohort with positive blood alcohol content presented IL-6 positive neurons and glial cells in comparable numbers to the rest of the TBI cases without alcoholic influence.

Neuronal IL-6 immunopositivity might be useful for clarifying the cause and timing of traumatic deaths when whole brain pathologies are taken into consideration, given the significant chronological changes in both the PCZ and the cerebellum in longer survival times.

### GFAP positive astrocyte numbers predict TBI survival time categories

GFAP is the principal marker for mature and differentiated brain astrocytes^65^ and is a major integral component of the astrocyte cytoskeleton^66^. Immediately after brain damage, GFAP is released from degenerating brain cells into the extracellular spaces and surrounding interstitial fluid^66^. Following the TBI associated brain tissue damage (such as hypoxia, ischemia or necrosis), the astrocytes become reactive and rapidly produce GFAP, to maintain the integrity of the CNS cells^26,67^. This agrees with the results of the PCZ with increasing GFAP positive astrocytes after two hours survival. The increase in GFAP positive astrocytes is most commonly associated either with a dissociation of glial filament bundles caused by a cellular edema, or an increase in GFAP synthesis due to an astrocyte fibrosis^68^.

A potential influence of anthropometric data with the GFAP levels was previously described in both animal and human studies^69,70^. This study showed a sex dependency of the GFAP positive astrocytes in control cases, which has not been described before^71^. The reason for this remains unclear. Also, the observed age influence on GFAP positive astrocytes was not described before in forensic settings^7,26,72^.

GFAP was previously investigated by immunohistochemical reactions in the PCZ in animals and humans. The protein positively labels activated glial cells next to cerebral contusion zones after one day of TBI survival at the earliest^25^ and reaching its maximum at the fourth posttraumatic day^73^. Increased GFAP expressions appeared even after 6 h post-TBI in single cases^6^. An astroglial response in cortical lesions after survival of three to five days has been described in rats before^74,75^. According to a recent post-mortem study, GFAP showed a significant increase in expression within three days survival period compared to acute TBI cases and controls^76^. Our study is in line with those findings.

Hausmann *et al*.^25^ investigated perifocally-distributed GFAP positive astrocytes adjacent to cortical contusions with the here used antibody. Comparing the microscopic fields investigated by their study and ours, the here proposed threshold value of 106 GFAP positive cells in the PCZ was reached not earlier than after three to four days of survival. Moreover, the threshold was not reached in the cortical zones of non-TBI controls in a former forensic study^25^, confirming the usability of our threshold calculations.

Despite the known reservations and arguments against ‘scoring systems’ or ‘gradings’ in histopathology and immunohistochemistry especially due to its largely subjective nature, the comparison between quantitatively-counted GFAP positive astrocytes and the qualitative statement about the staining intensity showed overall moderate to strong positive correlations. This finding indicates that a higher GFAP positive astrocytes count is associated with stronger marker enrichment. Regarding the underlying four-grade scoring, this result seems to be conclusive. The antibody used in this study reacts strongly with human GFAP, whose expression increases with the (re-)activation of astrocytes to extend their processes and subsequently unfolds abilities like stabilization of the CNS integrity after TBI. Thus, a stronger staining and higher number of stain positive astrocytes would indicate high levels of astrocytic differentiation and activity, characteristic of astrogliosis^35^. Despite its subjective nature, this result also supports the use of the grading system for GFAP.

Interestingly, serum GFAP values gradually reduce from the onset of trauma to the third day post injury in living patients^66^. Similarly, in previous post-mortem biochemistry investigation using cadaveric fluids in delayed TBI death cases also showed the lowest GFAP levels in the cerebrospinal fluid^14^. This reciprocal behavior of decreasing peripheral GFAP amounts in body fluids and increasing GFAP expression in astrocytes in the CNS, substantiate the existence of a local astrogliosis starting at the third post-traumatic day at the latest as part of continued secondary brain changes after TBI in terms of neuroregeneration^77^. This is based on the mechanism that activated astrocytes may protect the surrounding brain cells (‘traumatic penumbra’) from secondary lesions^78^. This may promote healing and functional recovery of neurophysiologic pathways^79^. Glial swelling was described to be one of the main mediators of diffuse brain edema^80^ and starts after a TBI^81^. This could be one of the reasons for the increasing numbers in GFAP positive astrocytes with increasing survival time. Given that histological signs of brain swelling due to cerebral edema are of higher validity than gross brain features in forensics, the necessity for histological examinations in any brain death autopsy case is underlined^82^. This should also include immunohistochemistry to estimate the survival time in the case of TBI from our perspective.

Halliday *et al*.^65^ described differences in the pattern of GFAP staining when using different antibodies, which may account for discrepancies between this study and previous immunohistochemical studies^7,26^ as different antibodies might detect reactive changes to different degrees.

Conclusively, a GFAP negative cell staining might not necessarily indicate that this cell is of non-astrocytic origin (although it was formerly stated that GFAP should theoretically be contained in all astrocytes)^83^, which might have practical legal implications for the results reported.

The CA4 region of the hippocampus has been described as a hypoxia-sensible part of the hippocampus^7^, forming the rationale to compare this area in our study to other brain regions. Here, no statistically relevant changes in the staining patterns of IL-6 in neurons as well as of GFAP in astrocytes were found in our study. Other components of the hippocampal formation might show other positively stained cell numbers, as has been shown before for the CA1 region with the application of two other neuro-biomarkers (S100 and neuron-specific enolase)^8^. However, three Japanese studies described a decrease of GFAP positive astrocytes in the CA4 region of the hippocampus in longer survival periods^7,26,71^. In contrast, we did not demonstrate significant changes but a trend to higher astrocyte numbers in delayed TBI deaths.

So far, only two papers have investigated brain regions other than the cortical PCZ and the hippocampus in human TBI: the thalamus, hypothalamus, striatum^84^ as well as the corpus callosum and the cerebellum^85^. There were overall higher GFAP positive cells in these structures after TBI compared to controls but these regions were not investigated in our cohort, except the cerebellum. To our best knowledge, only Crooks *et al*.^85^ published about GFAP immunohistochemistry in the cerebellum. Our results indicate that there were no relevant changes in the cerebellar numbers of GFAP positive astrocytes after traumatic impact. However, a GFAP immunohistochemistry might be useful especially in brain samples of the brain cortical areas and will result in limited information when investigated in regions further away from the brain cortex. The trend of increasing GFAP positive cell numbers in the PCZ and the contralateral side on a protein level is further substantiated by the results of gene expression analyses where the GFAP mRNA in these two localizations were increased already after subacute TBI survival times^15^.

### Limitations

Similar to previous immunohistochemical counting studies, we stress that evaluating routine histological staining (H&E) is necessary to evaluate the quality of the tissue specimens like it was stated before^65,71,82^. The proposed thresholds can be applied when using the same antibodies and dilutions, counting methods and brain areas as described and they cannot be generalized to other pre-analytical methods. To be accurate, the tissue collection of PCZ samples should include a tissue seam of at least 5 mm relative to the injury site, including adjacent cortical layers and white matter. Fortunately, the brain tissue samples from forensic autopsies are generally cubes of 1 cm³ and, therefore, much larger than required. It was reported before, that there are differences in the staining between monoclonal and polyclonal antibodies^26^, given that the latter one may recognize a higher epitope number^65^. To overcome this problem, we have decided to use commercially available polyclonal antibodies only in our routine and research consistently.

Further, both proteins are known to be non-specific TBI responders in the CNS^21,45^, rendering the interpretation of stained sections of uninjured brain tissue potentially difficult. The PCZ is therefore the most promising brain area to be investigated for reliable statements in intracellular IL-6 and GFAP staining changes.

Although GFAP can potentially degrade during longer storage conditions^10^, PMI had no relevant influence on the staining for up to six days in our study. With the PMI being a common confounding factor in post-mortem examination, the presented immunohistochemical and immunofluorescence methods suggest that GFAP staining was unaffected by PMI, and therefore seem to be useful for forensic pathologists for their daily work.

Although the case – control selection had a strict exclusion criteria, there was a statistical difference in the age between the two groups. This is explained by the differences in the onset of life-threatening cardiovascular diseases in later years and the typical age distribution of trauma deaths with special focus on severe TBI (accompanied by a minutely survival chance, “young risk takers”) in younger years of life^1^.

## Conclusion

A systematic chronological change of intracellular IL-6 and GFAP positivity in neurons and glial cells has been shown, depending on the trauma survival time especially in the PCZ. We conclude that higher numbers in IL-6 and GFAP stained cells above the threshold values in the PCZ substantiate the existence of potentially fatal traumatic changes within the CNS with reasonable certainty. Post-mortem immunohistochemistry has great potential to complement and further objectify the survival time estimation in the daily routine in legal TBI investigations, especially when ante-mortem data are lacking or absent.

## Methods

### Collection and stratification of samples

This research study has been approved by the ethics committee of the medical faculty of the University of Leipzig, Germany (local number 117-12-23012012). Following the approval of the ethical committee, the next of kin of the deceased (when personally known) or the public prosecutor’s office Leipzig (if no relatives were available) gave their informed consent to analyze the brain tissue samples, which were anonymized according to the guidelines from the central ethic commission of the federal medical association. All methods were carried out in accordance with the relevant guidelines and regulations.

This study covered autopsy cases from the Institute of Legal Medicine of the University of Leipzig between 2010 and 2016 (inclusive). The analyses formed part of an ongoing project assessing selected contusion zones in TBI cases following a similar protocol. Post-mortem brain tissue samples next to macroscopically or microscopically verifiable cortex contusions (cortical hemorrhages with abundant necrotic tissue) were collected as pericontusional zone (PCZ). Samples were than taken into neutral buffered 4% formaldehyde for fixation in non-penetrating blunt TBI cases. The exact survival time was known from the medical records or police investigations, which ranged between several minutes to 145 days. Of these TBI cases, additional brain samples were stored identically in small tissue cubes of about 1 cm³ to gather information of the time course of generalized (secondary) changes in the CNS such as hypoxia or ischemia located away from the PCZ; these cortex samples included samples of the contralateral cortex, defined as the cortical area contralateral to the PCZ region in a coronial brain preparation being uninjured macroscopically and microscopically (absence of contre-coup bleedings), as well as samples of the ipsilateral hippocampus and the ipsilateral cerebellum, like done by our research group previously^8,10,15^. The workflow for the selection or exclusion of cases for this study is presented in Supplemental Fig. 1. The period of fixation varied between one and three weeks before samples were embedded in paraffin. To avoid errors during fixation, the formaldehyde solution was changed after 24 hours once.

The trauma cases were subdivided into three groups according to their individual trauma survival time being acute deaths after TBI (survival time < 2 h), subacute deaths after TBI (survival time 2 h–3 days) and delayed deaths after TBI (survival time > 3 days) in accordance to former studies^7,12–14^.

Inclusion criteria for brain tissues of control cases were defined as sudden natural cardiovascular causes of death, the absence of former or acute CNS injuries or diseases and a PMI matched collection compared to the TBI cases. For the control group, brain tissue was taken from the frontal cortex, the hippocampus and the cerebellum from one hemisphere.

The PMI was defined as the time span between the estimated time of death and tissue storage during autopsy and was set to a maximum of six days post-mortem without interruption of adequate cooling of the corpses in cooling chambers in the departmental morgue.

### Histological and immunohistochemical staining

Serial sections of the paraffin blocks were produced with a thickness of 6 μm. These sections were deparaffinized in xylene-substitute Neo-Clear for immunohistochemistry (Neo-Clear for immunofluorescence; Merck KGaA, Darmstadt, Germany) and in alcohol and were then rehydrated and stripped on SuperFrost^TM^PLUS glass slides (Thermo Fischer Scientific Inc., Waltham, United States). The tissue sections were firstly stained using hematoxylin and eosin to evaluate the morphology and possible traumatic or inflammatory changes.

The commercially available antibodies used for this given study were polyclonal rabbit anti-IL-6 (catalogue number 21865-1-AP; Proteintech Group, Rosemont, USA) with a dilution of 1:50 and polyclonal rabbit anti-GFAP (catalogue number Z0334; Agilent Dako, Santa Clara, USA) with a dilution of 1:4000.

Immunohistochemical staining was performed using the fully automated immunostainer Benchmark XT (Roche, Basel, Switzerland). Staining protocol included deparaffinization and counterstain with hematoxylin and blueing reagent according to the manufacturers´ instructions. The IL-6 antibody required pretreatment by microwave heating for twenty minutes, in citrate buffer at pH 6.0 (Agilent Dako). The sections were immersed for ten minutes in peroxidase blocking solution (Agilent Dako) to block the endogenous peroxidase. Pretreatment of slides was not necessary for GFAP staining. To verify the findings and to confirm antibody specificity, positive and negative controls were prepared consequently for every staining charge.

### Morphological assessment and semi-quantitative counting

Microscopic evaluation was conducted using the Carl Zeiss Axiolab optical microscope (magnification, 50x–400x). Cytological classification of cells as glial cells or neurons was performed according to established morphological criteria, like morphology of the nucleus, cell borders, and the location of the nucleolus. Cells were counted in the layers I-V of the cortical cortex and the white matter in the contralateral cortex and surrounding the direct tissue impact in PCZ, the CA4 region of the hippocampus and the cortex and medulla of the cerebellum. True to the idea of establishing practical knowledge for a routine forensic use of both markers, routine TBI autopsy samples with their typical disadvantages of cadaveric material was used. In order to ensure the highest possible reliability of the results, we only counted cells showing a distinct cellular staining which could be identified with certainty to one cell type. Evaluation was performed using the commercially available object counting application SIGMA (open source; developed by Karen Grigoryan in 2016) in ten digital images at 200x magnification (standard 10x ocular lenses and 20x objective lens) to ensure a ‘close-to optical high-power field image’ as described before^86^. The area investigated referred to 2.37 mm^2^ per brain region and case (for calculation see Trautz *et al*.^86^).

**Figure 8:**
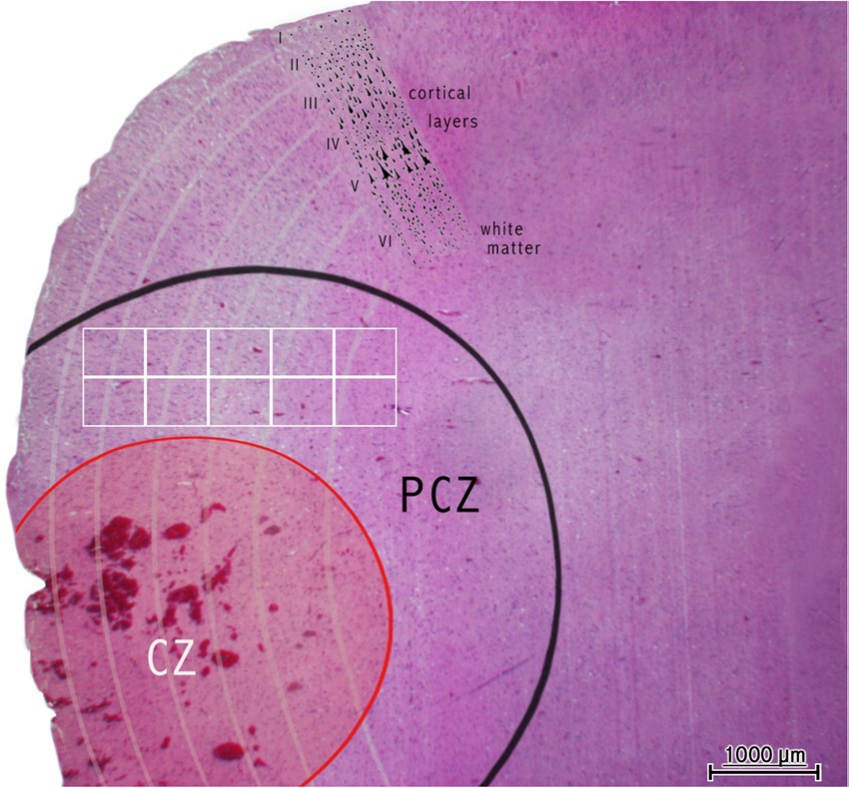
Example of the spatial relationship between the contusional zone (CZ, red circle) and pericontusional zone (PCZ, black circle) in a traumatic brain injury death case with illustrated cortical layers (grey hemicycles) and white matter. The white boxes within the PCZ illustrate an area of investigation (sum of 10 high power fields) as used in this study. Hematoxylin eosin staining, scale bar: 1000 μm.

The sampling method of PCZ depends on its size and location. The PCZ surrounding the contusional zone has an approximate distance up to 500 µm^22^ and the area investigated here equals 2.37 mm². Depending on the investigated cell population and their anatomical location in the CNS, a tissue seam of at least 5 mm relative to the injury site – including cortex and white matter – should be gathered to enable the possibility of detecting vivid IL-6 and GFAP positive cells. Figure 8 illustrates the underlying dimensions and one exemplified area of counting within the PCZ.

The ratio of IL-6 positive neurons were evaluated as total number of positive neurons/total number of all neurons. Given the minutely stained IL-6 glial cells in comparison to their overall appearance within the selected brain regions, the sum of positive numbers of astrocytes and oligodendrocytes were counted as numbers of glial cells in the PCZ and compared quantitatively. Further, the GFAP-positive numbers of astrocytes were counted and compared identically. Additionally, the staining intensity of GFAP positive astrocytes was rated and classified into grades (0) to (3) for each individual HPF. We here used a scoring system adapted to Remmele and Stegner^87^: grade (0) was defined as absence of markedly staining, grade (1) as light marker enrichment mainly concentrated on cell bodies, grade (2) as moderate staining occasionally accompanied by more intensively colored spots and some blunt processes and grade (3) as strong marker expression with predominately condensed central granulation and widely spread processes (see Supplemental Fig. 3 for visualization of the different grades). Following this, the arithmetic mean for 10 HPFs was accumulated for final grading of the brain tissue sample in question. The grading was then set in correlative comparison to the quantitative counting results.

### Immunofluorescence evaluation and confocal microscopy

For immunofluorescence labeling using paraffin embedded tissue sections the treatment procedure was performed as previously described^8,22,88^. In sum, after deparaffinizing and buffering, the slides were incubated with a mixture of the rabbit IL-6 antibody (see above) and mouse anti-MAP2 (microtubule-associated protein 2, 1:200; Millipore, Temecula, USA) as well as mouse anti-GFAP (1:1000; Sigma-Aldrich, St. Louis, USA) in 5% fetal calf serum in buffer over night at 4 °C. The primary antibodies were visualized with Cy2-conjugated donkey anti-rabbit (1:400) and Cy5-conjugated donkey anti-mouse IgG (1:200; both Jackson ImmunoResearch, West Grove, USA) after 2 h incubation. Further, slides were stained with Hoechst 33342 (1:1000, Molecular Probes, Leiden, Netherlands) to identify the cell nuclei by auto-fluorescence utilizing an ultraviolet laser (362 nm). Strict internal control runs were done without primary antibodies.

The immunofluorescence was investigated by a confocal laser scanning microscope (Leica SP8 confocal microscope; Leica, Wetzlar, Germany) using excitation wavelengths of 488 nm (argon laser, yellow-green Cy2-immunofluorescence labelling), 543 nm (helium/neon1, red Cy3-immunofluorescence) and 633 nm (helium/neon2, blue Cy5-labelling).

### Statistical analyses

Data analysis was conducted using Prism version 8 (GraphPad Software, La Jolla, USA) and Microsoft Excel version 16.15 (Microsoft Corporation, Redmond, USA).

After checking the data for normality using Shapiro-Wilk tests, we ran non-linear evaluations with Kruskal-Wallis tests for >2 groups comparisons and Mann-Whitney-U tests for calculations between two groups. For statistically significant results, *post-hoc* tests investigating significance of survival times according to Dunn’s multiple comparisons test were done using statistical hypothesis testing to avoid type I error accumulation. Adjusted p-values of 0.05 or less were considered as statistically significant. All correlations presented were computed using Spearman’s correlation coefficients using the same significance level. ROC curve analysis was used to identify the sensitivity and specificity of threshold values for the differentiation between TBI and controls by conservative estimations.

## Supplementary information

Dataset 1

## Data Availability

All data generated or analyzed during this study are included in this published article and its Supplementary Information Files.
