# Supplementary material for: Survival-time dependent increase in neuronal IL-6 and astroglial GFAP expression in fatally injured human brain tissue
Source: Sci Rep. 2019 Aug 15;9:11771. doi: 10.1038/s41598-019-48145-w (PMC6695416; doi:10.1038/s41598-019-48145-w)
Supplement: Supplementary file 1 — Dataset 1 [file 41598_2019_48145_MOESM1_ESM.pdf]

Survival-time dependent increase in neuronal IL-6 and astroglial GFAP expression in fatally injured human brain tissue

Florian Trautz[1,#], Heike Franke[2,#], Simone Bohnert[3], Niels Hammer[4-6], Wolf Mueller[7], Ruth Stassart[7], Rexson Tse[8], Johann Zwirner[4], Jan Dreßler[1], Benjamin Ondruschka[1,*]

[1] Institute of Legal Medicine, Medical Faculty University of Leipzig, Leipzig, Germany
[2] Rudolf Boehm Institute of Pharmacology and Toxicology, Medical Faculty University of Leipzig, Leipzig, Germany
[3] Institute of Forensic Medicine, University of Würzburg, Würzburg, Germany
[4] Department of Anatomy, University of Otago, Dunedin, New Zealand
[5] Department of Orthopedic and Trauma Surgery, University Hospital of Leipzig, Leipzig, Germany
[6] Fraunhofer IWU, Dresden, Germany
[7] Department of Neuropathology, University Hospital of Leipzig, Leipzig, Germany
[8] Department of Forensic Pathology, LabPLUS, Auckland City Hospital, Auckland, New Zealand

# Both authors contributed equally to this work.

[*] **Corresponding Author:** PD Dr. med. Benjamin Ondruschka
Medical Faculty University of Leipzig
Institute of Legal Medicine
Johannisallee 28
D-04103 Leipzig, Germany
phone: +49 (0) 0341-9715152
fax: +49 (0) 341-9715109
mail: benjamin.ondruschka@medizin.uni-leipzig.de

Electronic supplementary material:

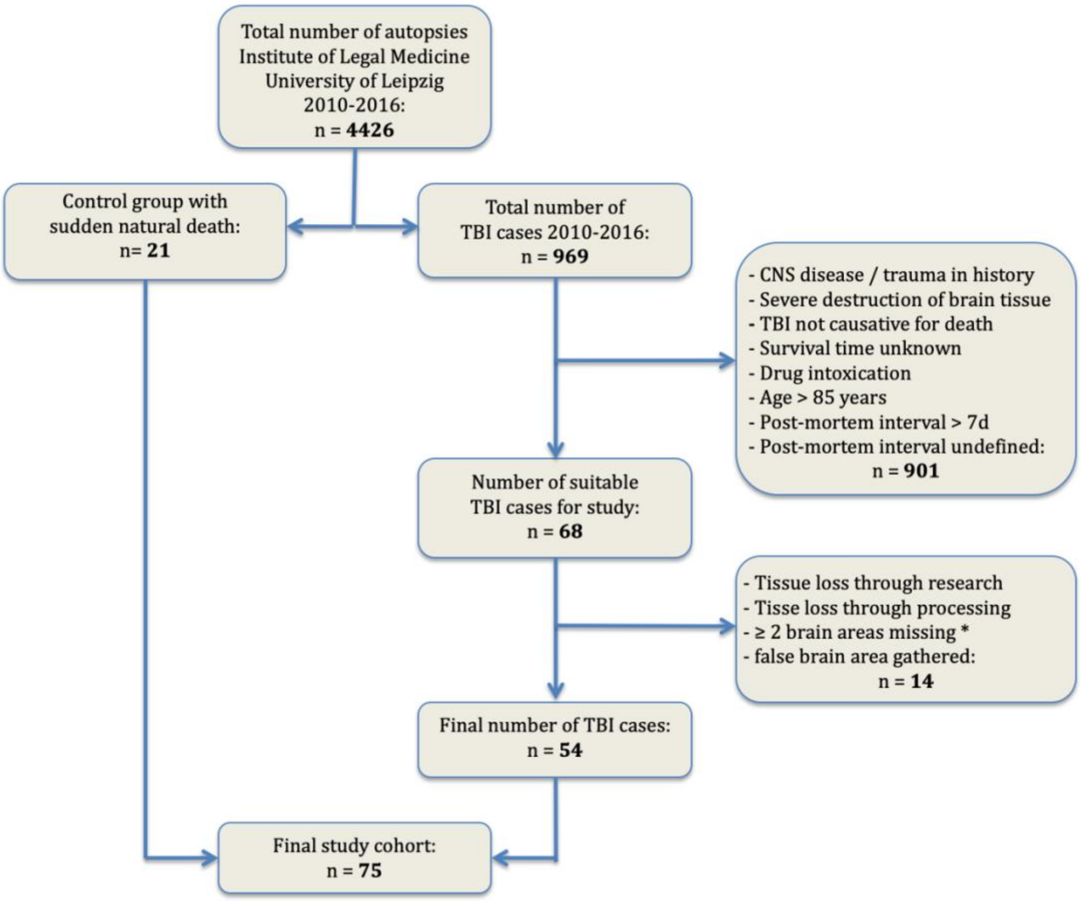

Supplemental figure 1: Workflow for traumatic brain injury study case selection out of the total of autopsies done at the Institute of Legal Medicine of the University of Leipzig in a 7-year timeframe.

CNS, central nervous system; TBI, traumatic brain injury; *, TBI cases were excluded for analysis if not at least two brain regions out of the contralateral cortex, hippocampus or cerebellum were available next to the pericontusional zone per case.

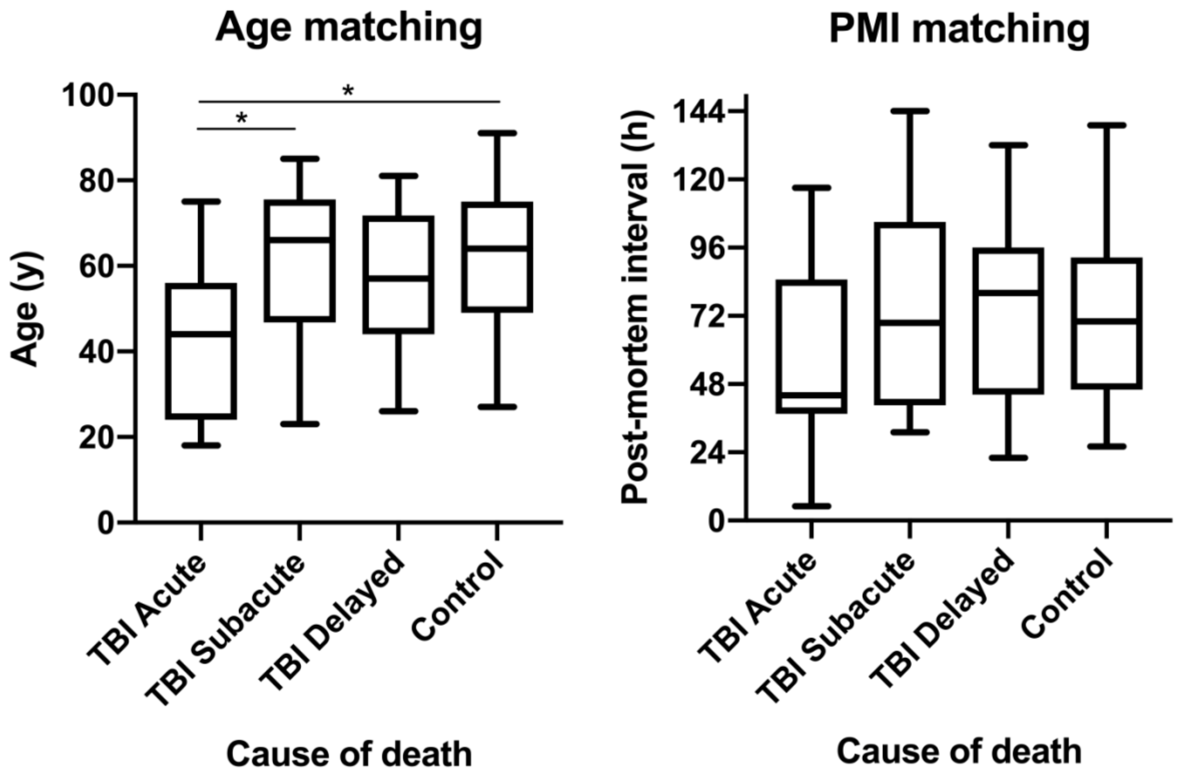

Supplemental figure 2: Box plot diagrams displaying the comparison between age of the deceased (left) and post-mortem interval (PMI, right) of the cases in respect to the survival time and cause of death. The outlines of the boxes indicate the 25% and 75% percentile, the solid black line the median. End of lines show the minima and maxima.

TBI, traumatic brain injury; y, years, h, hours; *, $p<0.05$ using Kruskal-Wallis test followed by *post hoc* Dunn's test.

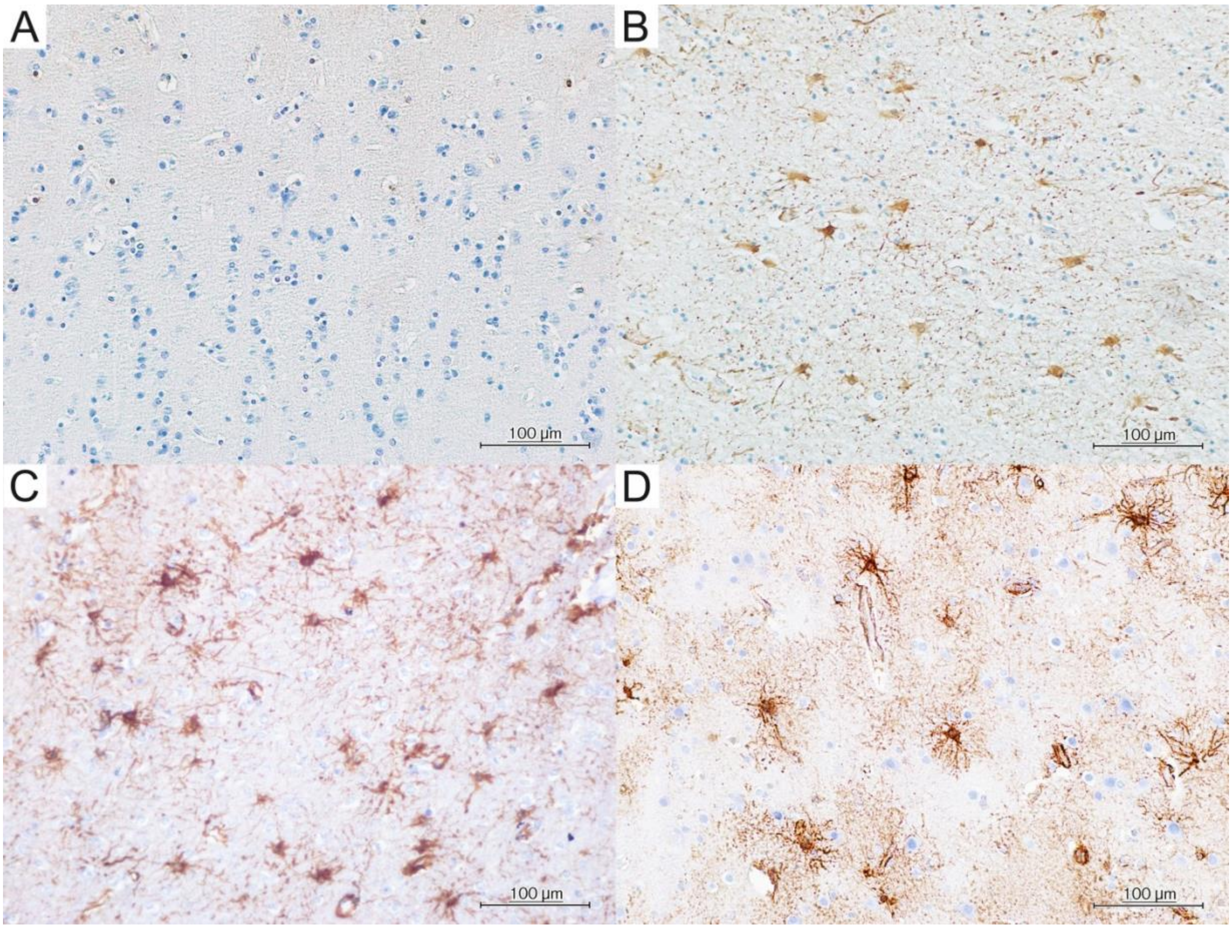

Supplemental figure 3: Different grades of staining intensity of GFAP positive astrocytes with exemplified pictures for grade 0 – absent staining (A); grade 1 – light marker staining on cell bodies (B); grade 2 – moderate staining with some blunt processes (C) and grade 3 – strong marker expression with widely spread processes (D). Scale bars: 100 µm.
